# Hand hygiene knowledge in long-term care facilities (LTCFS): a multicentre pilot study

**DOI:** 10.1186/1753-6561-5-S6-P163

**Published:** 2011-06-29

**Authors:** D De Wandel, D Vogelaers, S Blot

**Affiliations:** 1 Faculty of Health care, University College Ghent, Belgium; 2 Faculty of Medicine and Health Sciences, Ghent University, Belgium; 3 Department of General Internal Medicine and Infectious Diseases, Ghent University Hospital, Ghent, Belgium

## Introduction / objectives

Until now, promotion of hand hygiene was mostly targeted to acute care facilities. Yet, little is known about knowledge of hand hygiene recommendations in chronic care facilities. The objective of this study wasÂ to evaluate hand hygiene knowledge in health care workers in Flemish LTCFs.

## Methods

We developed a questionnaire based on campaign material that was distributed by the Flemish government in order to improve hand hygiene in LTCFs. The quiz, based on the most recent WHO guidelines contains 18 specific healthcare related situations in which hand hygiene or wearing gloves is recommended. It was presented to a stratified sample of personnel from two LTCFs in January 2011, prior to continuing education regarding hand hygiene. Frequencies and total scores were calculated. Mann-Whitney test was used to compare scores in both LTCFs. Statistical analyses were performed using PASW Statistics 18 (SPSS, Chicago, IL, US).

## Results

81 LTCF health care workers participated. Response was 100%. The mean score was 9.21 on 18 items (Standard Deviation 2.58, min. 1, max. 15) or 51%. No significant differences were found between the two LTCFs. Some items scored below 50%. These items were related to four hand hygiene indication groups: ‘before patient contact’ (e.g. hygienic care, taking blood pressure, feeding), ‘after glove removal’, ‘before a clean/aseptic procedure’ (e.g. wound care and IM injections) and ‘in case of risk of contact with body fluids’ (e.g. oral medication administration).

## Conclusion

Our study identified several gaps in the knowledge about hand hygiene recommendations in LTCF health care workers. These results indicate substantial room for improvement in this specific group of HCWs.

## Disclosure of interest

None declared.

